# Phenotypic and Molecular Characterisation of Extended-Spectrum Beta-Lactamase Producing *Escherichia coli* Obtained from Animal Fecal Samples in Ado Ekiti, Nigeria

**DOI:** 10.1155/2015/497980

**Published:** 2015-08-31

**Authors:** Olugbenga Adekunle Olowe, Olufunmilayo Adewumi, Gbolabo Odewale, Olusola Ojurongbe, Olusolabomi Jose Adefioye

**Affiliations:** ^1^Department of Microbiology and Parasitology, Ladoke Akintola University of Technology, Ogbomoso, Nigeria; ^2^Department of Medical Laboratory Science, Afe Babalola University, Ado Ekiti, Ekiti State, Nigeria

## Abstract

Production of extended-spectrum *β*-lactamases (ESBLs) producing* E. coli* in animals and different methods of identifications from Ado Ekiti, Ekiti State, Nigeria, were investigated. Three hundred and fifty fecal samples, collected from apparently healthy cattle and pigs, were cultured and identified following standard procedures. ESBL phenotypic detection was carried out using combination disc test, double disc synergism test, and ESBL brilliance agar screening. Molecular detection of TEM, SHV, and CTX-M genes was carried out using standard molecular method. One hundred and fourteen* E. coli* isolates were recovered from the 350 samples processed, out of which 72 (63.2%) isolates were positive for ESBLs with multiple resistance to the antibiotics used. Eighty-one (71%) isolates were positive for ESBL by combination disc test, 90 (78.9%) were positive for double disc synergism test, and 93 (81.6%) were positive for ESBL brilliance agar. TEM and CTX-M genes were detected in 48 (42.1%) and 51 (44.7%) isolates, respectively. SHV gene was not detected in any of the isolates while TEM and CTX-M were detected in 33 (28.9%) isolates. This study showed high resistance of* E. coli* to antibiotics, particularly to the third generation cephalosporins. Regular monitoring and regulated use of antibiotics in livestock should be encouraged.

## 1. Introduction

Production of extended-spectrum *β*-lactamases (ESBLs) is the most common mechanism of resistance to third-generation cephalosporins among Enterobacteriaceae including* Klebsiella pneumoniae* and* Escherichia coli* [1, 2]. ESBL determinants have been detected not only in clinical isolates but also in commensal bacteria from humans and animals and in isolates from products of the food chain and sewage, revealing distribution and suggesting the presence of environmental reservoirs for these resistance determinants [3, 4].

The increase in antimicrobial-resistant bacteria of animal origin resembles the process in humans about two decades ago [5]. Since the late 1990s, extended-spectrum *β*-lactamase (ESBL) producing Enterobacteriaceae, in particular* E. coli*, have emerged globally. Initially, ESBL producing bacteria were only observed in human medical practice [6, 7] but the recent observation of these bacteria, first in companion animals and increasingly in livestock, has initiated monitoring studies focused on livestock [8]. ESBL producing* E. coli* isolates are now being found in increasing numbers of food-producing animals leading to the hypothesis that animals might become infection sources or even reservoirs (the natural persistent source of infection) contributing to the spread of these bacteria [9].

Currently, there is paucity of information on ESBL producing* E*.* coli* from animals and the possible contribution of these resistant species to the ever growing antimicrobial resistance observed in humans. The broad objective of this study was to determine the occurrence of ESBL producing* Escherichia coli* in cattle and pigs in Ado Ekiti, South Western Nigeria.

## 2. Materials and Methods

### 2.1. Sample Collection

Three hundred and fifty fecal samples were collected from cattle (*n* = 200) and pigs (*n* = 150). Samples from apparently healthy cattle were collected from the colon immediately after slaughtering the animal at the abattoirs aseptically. Freshly passed feces from apparently healthy pigs were collected into sterile capped universal bottles with sterile spatula and were transported to the laboratory immediately.

### 2.2. Isolation and Identification

Isolates were recovered from the samples after culturing on modified selenite F and incubated for 18–24 hours at 37°C. The overnight incubated liquid selective media were inoculated on Sorbitol Macconkey Agar, Eosin Methylene Blue Agar, and Macconkey Agar and incubated for 18–24 hours at 37°C. Presumptive characteristic* E. coli* isolates were identified and confirmed using arrays of biochemical tests and API 20 E multitest systems (bioMérieux, France). The study was carried out in the Department of Medical Laboratory Science, ABUAD, Ado Ekiti, and the Department of Medical Microbiology and Parasitology LAUTECH, Osogbo, between February and November 2014.

### 2.3. Susceptibility Test

Antimicrobial susceptibility of pure colonies to Ampicillin (10 *μ*g), Amoxicillin (25 *μ*g), Augmentin (AUG) (30 *μ*g), Cefotaxime (CTX) (30 *μ*g), Ceftazidime (CAZ) (30 *μ*g), Cefuroxime (CXM) (30 *μ*g), Ciprofloxacin (CPX) (10 *μ*g), Cefixime (CXM) (5 *μ*g), Cefpodoxime (CP) (10 *μ*g), Ofloxacin (5 *μ*g), Imipenem (10 *μ*g), Tetracycline (30 *μ*), Gentamicin (GEN) (10 *μ*g), Streptomycin (STR) (10 *μ*g), Erythromycin (ERY) (10 *μ*g), Chloramphenicol (CHL) (25 *μ*g), Cloxacillin (5 *μ*g), Nitrofurantoin (NIT), and Cotrimoxazole (STX) (25 *μ*g) was determined and interpreted by the disc diffusion method following the guidelines of Clinical Laboratory Standard Institute [10].

### 2.4. Detection of Extended-Spectrum *β*-Lactamase (ESBL)

#### 2.4.1. Cefpodoxime and Cefpodoxime-Clavulanate (Combination Disc Screening)

Cefpodoxime and Cefpodoxime-clavulanate (Oxoid, Basingstoke, UK) discs were applied on freshly prepared Muller Hinton agar and the plates were incubated aerobically at 37°C. A final measurement of the zone of inhibition was made after overnight incubation. ESBL producers were defined as having a differential zone diameter of ≥+5 mm (Cefpodoxime-clavulanate zone − Cefpodoxime zone).

### 2.5. Double Disc Diffusion Test

A Ceftazidime 30 *μ*g disc and Cefotaxime 30 *μ*g disc with Amoxicillin/clavulanic acid 20/10 *μ*g (Oxoid, UK) were placed in the center 20 mm apart and incubated at 37°C for 18–20 hours [11]. ESBL production was inferred when the zone of inhibition around the Ceftazidime and Cefotaxime discs was expanded by the presence of clavulanate by ≥5 mm.

### 2.6. ESBL Brilliance Agar

Oxoid ESBL brilliance agar was inoculated according to manufacturer's instructions. Plates were inoculated with 1 *μ*L (a standard loopful) of a 0.5 McFarland standard suspension of* E*.* coli*. Subsequently they were incubated aerobically at 35 to 37°C for 18 to 24 hours in an inverted position and presumptive ESBL* E*.* coli* were identified based on color production.* Escherichia coli* produced blue/pink coloration following 24–48 hours of incubation. The reference* E*.* coli* strain ATCC25922 was inhibited and was used as negative control.

### 2.7. Polymerase Chain Reaction to Detect* bla* Genes

The DNA of the ESBL confirmed isolates was extracted separately using a DNA extraction kit (Biospin plasmid extraction, Bioflux, Japan). According to previously published work, PCR was used to detect *bla*_TEM_, *bla*_CTX-M_, and *bla*_SHV_ genes using specific primers [12]. Routine reactions were performed using the PCR master mix containing 3 *μ*L deoxynucleoside triphosphate (dNTPs), 4 *μ*L ×10 Tris EDTA buffer, 0.2 *μ*L* Thermus aquaticus* (Taq) polymerase, 0.5 *μ*L MgCl_2_, 5 *μ*L DNA lysates, 15.3 *μ*L of PCR water, and 1 *μ*L of forward and reverse primers in a 30 *μ*L final reaction volume. Electrophoresis was done using 1% Agarose.

### 2.8. Statistical Analysis

The sensitivities and specificities of the tests were determined. Differences in sensitivity and specificity of the tests were analyzed with the chi square test and log linear analysis using the Statistical Package for Social Sciences software (SPSS version 21).

## 3. Results

Table 1 shows the highest prevalence of* Klebsiella* spp. both in cattle and in pigs showing 125 and 70, respectively, while the least bacterial isolate was* Proteus* spp. with a prevalence of 21 and 10 for cattle and pigs, respectively, and* Escherichia coli* and* Pseudomonas* spp. were 114 and 129, respectively.

Table 2 shows the distribution and sources of fecal samples; female cow investigated was 73, while bull male was 127 and 10 large pigs were the least collected.

### 3.1. Antibiotic Susceptibility Patterns of* E. coli* Isolates

Table 3 shows the results of all the 114* E. coli* isolates that were subjected to antimicrobial testing and interpreted as resistant, intermediate, and sensitive following the guidelines of Clinical Laboratory Standard Institute [10]. The overall resistance of the isolates to antibiotics shows that resistance to Penicillin (PEN) (96%), Ampicillin (AMC) (89%), Amoxicillin (AMX) (88%), Augmentin (AUG) (96%), Cefotaxime (CTX) (92%), Cefuroxime (CPX) (83%), Cloxacillin (CXC) (84%), and Cotrimoxazole (STX) (90%) was high while resistance to Ciprofloxacin and Ofloxacin was low with 5% resistance to these antibiotics. The isolates also showed considerable resistance to Ampicillin, Tetracycline, Cotrimoxazole, and Cephalosporins as shown in Table 3.

The number of isolates that showed resistance simultaneously to twelve (12) different antibiotics was 42 (37.8%) and was highest among those isolates that demonstrated multiple antibiotic resistance while only 6 (5.6%) isolates showed resistance simultaneously to fifteen out of the 20 antibiotics tested. All the 114* E. coli* isolates showed resistance to at least nine antibiotics. All isolates that were resistant to more than two classes were identified as multidrug resistant (MDR) isolates and were selected for possible ESBL production screening (Table 4).

### 3.2. Phenotypic Test

**Figure 1:**
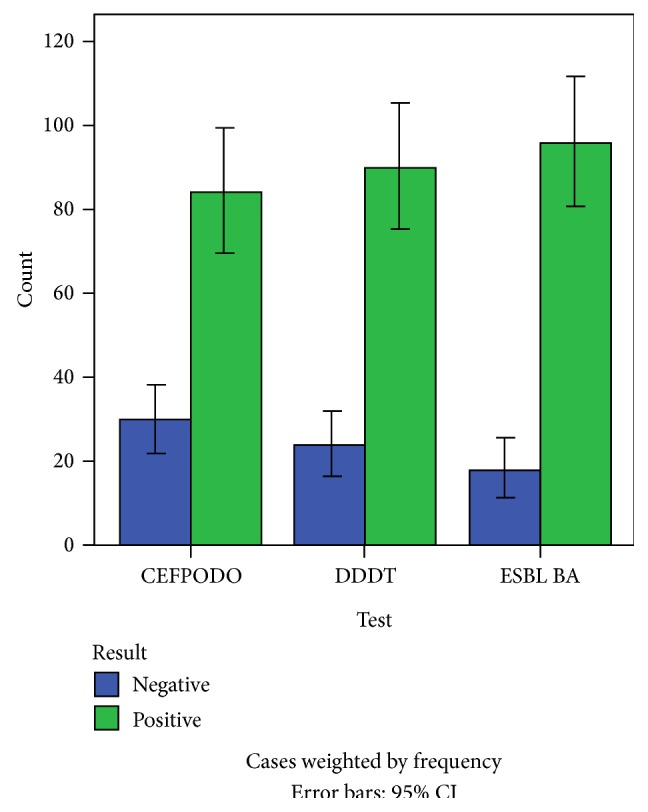
Representation of phenotypic detection of ESBL.

Figure 1 shows the results of the phenotypic tests used. Eighty-one (71%) isolates out of the 114 isolates were positive for combination disc test (Cefpodoxime/clavulanic acid) as shown in the figure. There was a significant association (*p* = 0.026) between Cefpodoxime/clavulanic acid and the detection of ESBL genes (CTX, SHV, and TEM). The sensitivity of the test was calculated using molecular detection as the gold standard was 89% while specificity was 82%. The rate of false positive prediction (1 − specificity) was 0.18 (18%). Double disc synergism test using Cefotaxime/Ceftazidime/clavulanate substrate was positive in 90 (78.9%) isolates out of the 114 isolates. There was an association between this phenotypic test and the detection of CTX-M genes (*p* = 0.025) and TEM genes (*p* < 0.001) (Table 5). The sensitivity of the test was 80% while specificity was 70%. The rate of false positive prediction (1 − specificity) was 30%. ESBL brilliance agar was positive in 93 (81.57%) isolates. There was a significant association (*p* < 0.001) in the result obtained by Cefpodoxime/clavulanic acid and ESBL brilliance agar. The sensitivity of the test was 79% and specificity was 67% while rate of false positive prediction was 33%.

### 3.3. Detection of ESBL Genes

The molecular characterization of resistant gene of the* E. coli* isolates using PCR was analysed. SHV showed no specific amplification and thus was not detected in any of the samples. A total of 51 (44.7%) isolates showed detectable CTX-M genes while TEM genes were present in 48 (42%)* E. coli* isolates.

## 4. Discussion and Conclusion

Antibiotic resistance has continued to constitute serious problems not only in human medicine but also in animal husbandry, livestock management, and veterinary medicine [13, 14].

In this study, several methods of detection were employed as previously reported methods of ESBLs detection. The major finding in the present study is the presence of multiple drug resistant commensal* E. coli* in animals to commonly used antibiotics such as Penicillin (96%), Augmentin (96%), Cefotaxime (92%), Ceftazidime (58%), Cefuroxime (83%), and Cotrimoxazole (STX) (90%). This observation reiterates the finding in other studies that have reported antibiotic resistance among bacteria especially* E. coli* isolated from cattle and other animals is increasing at an alarming rate [15–18]. In this study, susceptibility of all the isolates that showed multiple resistance to a minimum of nine antibiotics was 3 (2.6%). 12 (10.5%) isolates were resistant to ten antibiotics, 6 (5.6%) isolates were resistant to 13 different antibiotics, and 42 (36.8%) isolates were resistant to twelve antibiotics, while 9 (7.9%) isolates were resistant to fifteen antibiotics. This finding correlates with similar results obtained from other studies that have reported some levels of multiple antibiotic resistance by* E. coli* from cattle, meat products, and other animals [19, 20]. Ajayi et al. [17] determined the antibiotic susceptibility patterns of commensal* E. coli* from feces of apparently healthy cattle in Ado Ekiti, Nigeria, from ready to slaughter cattle. Their result revealed* E. coli* isolates which showed resistance to at least 3 of the eight antibiotics tested [17]. All the isolates showing multiple antimicrobial resistance in this study were screened for possible ESBL production. Extended-spectrum *β*-lactamase indeed is a superbug of trouble to clinicians and microbiologists and is creating environmental stress to pharmaceutical pipeline in the development of new antibiotics.

The Health Protection Agency of the United Kingdom recommends testing Cefpodoxime or both Cefotaxime and Ceftazidime as a first screening test [21]. This study revealed the combination of the two latter drugs separated at 20 mm distance achieves 80% sensitivity to adequately detect ESBL production, meaning that only 20% of the isolates would need further testing. This conforms to some other studies. Garrec et al. [22] reported a sensitivity of 77% using this same method. Generally, evaluations of the double disc diffusion test have revealed sensitivities of the method ranging from 79% to 97% and specificities ranging from 94% to 100% [23, 24]. The sensitivity of Cefpodoxime in this study was 89% while specificity was 82%. The rate of false positive prediction (1 − specificity) was 0.18 (18%). Cefpodoxime was found to have the highest sensitivity in this study and is thus in agreement with a study by Jain and Mondal [25] who concluded their report using the standard disc diffusion as screening test for identifying ESBL producers that Cefpodoxime was found to be the most efficient antimicrobial agent having a sensitivity of 93% and a specificity of 85.7% in screening isolates as potential ESBL producers followed by Ceftazidime with a sensitivity of 89.6% and specificity of 80.9% and Cefotaxime having sensitivity of 81.6% and specificity 85.7% [25]. However, it has been reported that it is adequate to use Cefotaxime, which is consistently susceptible to CTX-M, and Ceftazidime, which is a consistently good substrate for TEM and SHV variants but if only one drug is to be used, then the single best indicator for ESBL producer has been found to be Cefpodoxime [11, 26].

Using the Oxoid ESBL brilliance Agar, the sensitivity was 79% and specificity was 67%, while rate of false positive prediction (1 − specificity) was 33%. This was in contrast to a study published by Huang et al. [27], who reported a sensitivity of 94.9% and specificity of 95.7%. According to data on file at Oxoid (http://www.oxoid.com/), the performance of this product was rated as sensitivity having 95% and specificity having 94%. The differences observed in this study could be due to the use of already well characterized (known) ESBL producing isolates in the evaluation as well published that any results obtained by this method are presumptive and should be confirmed by other methods.

Following the screening of the* E. coli* isolates for ESBL genes using Polymerase Chain Reaction (PCR), *bla*_TEM_ was detected in 48 (42.1%) and *bla*_CTX-M_ was detected in 51 (44.7%) and *bla*_SHV1_ was not detected in any of the isolates. These findings suggest that *bla*_CTX-M_ was more common among the ESBL genes in these isolates which conforms to other studies [11, 28]. In this study, the occurrence of ESBL producing* E. coli* was as high as 63.2% as ESBL genes were detected in 72 out of 114 isolates screened. This is alarming and worrisome and higher than reports from clinical isolates in Nigeria, class A and D ESBLs and p-AmpC were found in hospital settings, and the prevalence ranged from 10.3 to 27.5% [6, 29–32].

Although most of these studies were done using* E*.* coli* isolates from humans, ESBLs have been reported to be plasmid encoded implying that these resistance determinants are found in our environment and can be transferred from one organism to another. The high prevalence of these ESBL producers in fecal samples of animal further buttresses the hypothesis that animals might become infection sources or even reservoirs (the natural persistent source of infection) contributing to the spread of these bacteria [9]. These resistant traits call for gross surveillance in community settings because ESBLs gene is a world growing treat for available antibiotics but its epidemiological effect and evaluation are still underestimated with low awareness.

In conclusion, to the best of our knowledge this is the first report of phenotypic and molecular investigation of* bla genes* (CTX, SHV, and TEM) from animal fecal samples and epidemiological relevance of different phenotypic and molecular methods. Multiple drug resistant ESBL producing* E. coli* is present in fecal samples of cattle and pigs in Ado Ekiti. The differences observed in the detection of ESBL positive isolates by the three different methods may be justified by the lower sensitivity of the phenotypic methods and the influence of environmental factors on the incidence of resistance.

## Figures and Tables

**Table 1:** Distribution of gram negative organisms isolated from cattle and pigs.

Number of samples	*Proteus *spp.	*E. coli*	*Pseudomonas *spp.	*Klebsiella *spp.
Cattle	21	79	84	125
Pigs	10	35	45	70
Total	31	114	129	195

**Table 2:** Distribution and sources of the fecal samples.

Cattle	Pigs
Cow (female) 73	Soar (female) 135 (10 piglets)
	Large white 65
	Large black 10
	Hampshire 45
	Duroc 15

Bull (male) 127	Boar (male) 15

**Table 3:** Antimicrobial sensitivity patterns of *E. coli* isolates based on the classes of antibiotics tested.

Class/antibiotics	Number of resistant isolates (%)	Number of intermediate isolates (%)	Number of sensitive isolates (%)
*β*-lactams			
Penicillin	110 (96%)	04 (4%)	—
Amoxicillin	100 (88%)	14 (12%)	—
Ampicillin	102 (89%)	12 (11%)	—
Augmentin	110 (96%)	04 (4%)	—
Ceftazidime	66 (58%)	27 (24%)	21 (18%)
Cefotaxime	105 (92%)	9 (8%)	—
Cefixime	45 (39%)	42 (37%)	27 (24%)
Cefuroxime	95 (83%)	10 (9%)	8 (7%)
Cefpodoxime	66 (58%)	06 (5%)	42 (37%)
Tetracycline	100 (88%)	9 (8%)	4 (4%)
Erythromycin	94 (82%)	15 (13%)	5 (4%)
Streptomycin	90 (79%)	15 (13%)	9 (8%)
Gentamicin	56 (49%)	03 (3%)	55 (48%)
Ciprofloxacin	6 (5%)	6 (5%)	102 (90%)
Ofloxacin	6 (5%)	5 (4%)	103 (90%)
Cloxacillin	96 (84%)	18 (16%)	—
Cotrimoxazole	102 (90%)	12 (10%)	—
Nitrofurantoin	—	12 (11%)	102 (89%)
Chloramphenicol	105 (92%)	9 (8%)	—
Carbapenem			
Imipenem	—	5 (4%)	109 (96%)

**Table 4:** The frequency distribution of antibiotics and resistant isolates.

Number of antibiotics	Number of resistant isolates (%)
09	03 (2.6%)
10	12 (10.5%)
11	06 (5.6%)
12	42 (37.8%)
13	24 (21%)
14	18 (15.8%)
15	09 (7.9%)

**Table 5:** Association of phenotypic tests and molecular characterization of *E. coli* isolates from animals.

Interactions	Chi square (*χ* ^2^)	Significance	Degree of freedom
CEFPODOXIME/ESBL BA/DDDT/GENE	0.000	1.00	1
ESBL BA/GENES	0.000	1.000	1
CEFPODOXIME/DDST/GENE	4.961	0.026^*∗*^	1
CEFPODOXIME/ESBL BA	17.540	<0.001^*∗*^	1
ESBL BA/DDST	24.466	<0.001^*∗*^	1
ESBL BA/CTX_M	0.000	1.000	2
CEFPODOXIME/DDST/CTX	5.009	0.025^*∗*^	1
ESBL BA/TEM	0.000	1.000	2
CEPODOXIME/TEM	0.165	0.684	2
DDST/TEM	22.407	<0.001^*∗*^	1
ESBL BA/SHV	0.000	1.000	2
DDST/SHV	0.000	1.000	2
CEFPODOXIME/SHV	0.000	1.000	2

^*∗*^Statistically significant.

CEFPODOXIME: Cefpodoxime screening test.

DDST: double disc synergism test.

ESBL BA: ESBL brilliance agar screening test.

SHV: detection of SHV genes.

TEM: detection of TEM genes.

CTX-M: detection of CTX-M genes.
